# Draft Genome Sequence of the Agar-Degrading Bacterium *Catenovulum* sp. Strain DS-2, Isolated from Intestines of *Haliotis diversicolor*

**DOI:** 10.1128/genomeA.00144-14

**Published:** 2014-03-06

**Authors:** Dapeng Shan, Xu Li, Zheng Gu, Guangshan Wei, Zheng Gao, Zongze Shao

**Affiliations:** a Key Laboratory of Marine Biogenetic Resources, Third Institute of Oceanography, State Oceanic Administration, Xiamen, People’s Republic of China; b State Key Laboratory of Crop Biology, College of Life Sciences, Shandong Agricultural University, Taian, Shandong, People’s Republic of China

## Abstract

*Catenovulum* sp. strain DS-2, isolated from intestines of *Haliotis diversicolor*, is able to degrade agar and produce agaro-oligosaccharides. Here, we report the draft genome sequence of *Catenovulum* sp. strain DS-2.

## GENOME ANNOUNCEMENT

*Catenovulum* sp. strain DS-2, isolated from the intestines of *Haliotis diversicolor*, is a Gram-positive, aerobic, nonmotile, mesophilic strain and is capable of degrading agar and producing agaro-oligosaccharides. Agar is composed of agarose and agaropectin (1). Agarose consists of linear galactans with a backbone of alternating 3-*O*-linked β-d-galactopyranose and 4-*O*-linked 3,6-anhydro-α-l-galactose units (2). Agarose can be specifically hydrolyzed by agarases (3). *Catenovulum* sp. DS-2 grows well in marine agar 2216E medium, and it can form a clear zone around the colony.

Here, we present the genome sequence of *Catenovulum* sp. DS-2, which was obtained using Solexa paired-end sequencing technology (4) by Shanghai Majorbio Bio-pharm Technology Co., Ltd. (Shanghai, China). A library with a fragment length of 300 bp was constructed, and a total of 4,347,955 paired-end reads were generated, resulting in a 154-fold depth of coverage, with an Illumina/Solexa Genome Analyzer IIx (5) (Illumina, SanDiego, CA). The gaps among the scaffolds (6) were closed by custom primer walks or by PCR amplification, followed by sequencing. The genome sequence of strain DS-2 comprises 4,572,520 bp, with an average G+C content of 40.71%, and it consists of 143 contigs (N_50_, 93,862 bp). Automatic gene annotation was carried out by the NCBI Prokaryotic Genomes Automatic Annotation Pipeline (PGAAP) (http://www.ncbi.nlm.nih.gov/genomes/static/Pipeline.html) and was followed by manual editing. The genome sequence contains 4,090 candidate protein-coding genes, giving a coding intensity of 89.9%, and the average size of each gene is 1,004 bp. A total of 1,038 proteins were assigned to Clusters of Orthologous Groups (COG) (7) families. In addition, 62 tRNA genes for 20 amino acids and one 16S-23S-5S rRNA operon were identified in the genome.

The genes possibly responsible for agarose degradation were analyzed in the genome sequence of strain DS-2. In total, 17 putative beta-agarases and 1 putative alpha-agarase were detected, which should fulfill the function of agarose degradation. Furthermore, there are 3 kappa-carrageenase and 3 iota-carrageenase genes in the genome, according to the annotation. The genome information and annotation reported in the present study are valuable for future research to investigate agarose degradation in marine environments.

### Nucleotide sequence accession numbers.

This whole-genome shotgun project has been deposited at DDBJ/EMBL/GenBank under the accession no. ARZY00000000. The version described in this paper is version ARZY01000000.

## References

[1] Rochas C Lahaye M Yaphe W 1986 ^13^C-N.M.R.-spectoscopic investigation of agarose oligomers. Carbohydr. Res. 148:199–207. 10.1016/S0008-6215(00)90388-4 8156559

[2] Duckworth M Yaphe W 1971 Structure of agar: part I. Fractionation of a complex mixture of polysaccharides. Carbohydr. Res. 16:189–197. 10.1016/S0008-6215(00)86113-3

[3] Day DF Yaphe W 1975 Enzymatic hydrolysis of agar: purification and characterization of neoagarobiose hydrolase and *p*-nitrophenyl α-galactoside hydrolases. Can. J. Microbiol. 21:1512–1518. 10.1139/m75-223 137

[4] Bentley DR Balasubramanian S Swerdlow HP Smith GP Milton J Brown CG Hall KP Evers DJ Barnes CL Bignell HR Boutell JM Bryant J Carter RJ Keira Cheetham R Cox AJ Ellis DJ Flatbush MR Gormley NA Humphray SJ Irving LJ Karbelashvili MS Kirk SM Li H Liu X Maisinger KS Murray LJ Obradovic B Ost T Parkinson ML Pratt MR Rasolonjatovo IM Reed MT Rigatti R Rodighiero C Ross MT Sabot A Sankar SV Scally A Schroth GP Smith ME Smith VP Spiridou A Torrance PE Tzonev SS Vermaas EH Walter K Wu X Zhang L Alam MD Anastasi C 2008 Accurate whole human genome sequencing using reversible terminator chemistry. Nature 456:53–59. 10.1038/nature07517 18987734 PMC2581791

[5] Farrer RA Kemen E Jones JD Studholme DJ 2009 *De novo* assembly of the *Pseudomonas syringae* pv. *syringae* B728a genome using. Illumina/Solexa short sequence reads. FEMS Microbiol. Lett. 291:103–111. 10.1111/j.1574-6968.2008.01441.x 19077061

[6] Boetzer M Pirovano W 2012 Toward almost closed genomes with GapFiller. Genome Biol. 13:R56. 10.1186/gb-2012-13-6-r56 22731987 PMC3446322

[7] Tatusov RL Galperin MY Natale DA Koonin EV 2000 The COG database: a tool for genome-scale analysis of protein functions and evolution. Nucleic Acids Res. 28:33–36. 10.1093/nar/28.1.33 10592175 PMC102395

